# Diversification of spiny-throated reed frogs (Anura: Hyperoliidae) with the description of a new, range-restricted species from the Ukaguru Mountains, Tanzania

**DOI:** 10.1371/journal.pone.0277535

**Published:** 2023-02-02

**Authors:** Lucinda P. Lawson, Simon P. Loader, John V. Lyakurwa, H. Christoph Liedtke

**Affiliations:** 1 Department of Biological Sciences, University of Cincinnati, Cincinnati, Ohio, United States of America; 2 Department of Zoology, Field Museum, Chicago, Illinois, United States of America; 3 Life Sciences Department, Natural History Museum, London, United Kingdom; 4 Department of Zoology and Wildlife Conservation, University of Dar es Salaam, Dar es Salaam, Tanzania; 5 Ecology, Evolution and Development Group, Department of Wetland Ecology, Estación Biológica de Doñana (CSIC), Sevilla, Spain; National Cheng Kung University, TAIWAN

## Abstract

The spiny-throated reed frog species group is a small radiation of *Hyperolius* frogs from East Africa. Unlike many members of the genus which have relatively wide distributions, these species tend to be small-range endemics found in montane and submontane forests. Recent discovery of a golden-hued frog with the clade-specific traits of spines on their gular discs prompted a morphological and genetic exploration of the distinctness of this new lineage and relationships to other members of the clade. Genetic (mitochondrial and nuclear loci) results resolved many sister-relationships, but deeper nodes in the phylogeny were poorly resolved. A reduced-representation genome-wide Single Nucleotide Polymorphism (SNP) dataset was able to fully resolve the phylogenetic relationships within this clade, placing this new lineage, here named after the mountain range in which is it found–*H*. *ukaguruensis* sp. nov., as an early diverging lineage within the group. This new species is distinct from all other spiny-throated reed frogs, necessitating further understanding as a single-mountain endemics vulnerable to habitat loss and potential decline. Morphometric analyses identify clear morphological characteristics that are distinct for the herein described species, most noticeably in that the eyes are significantly smaller than other members of the genus for which we have samples.

## Introduction

**Fig 1:**
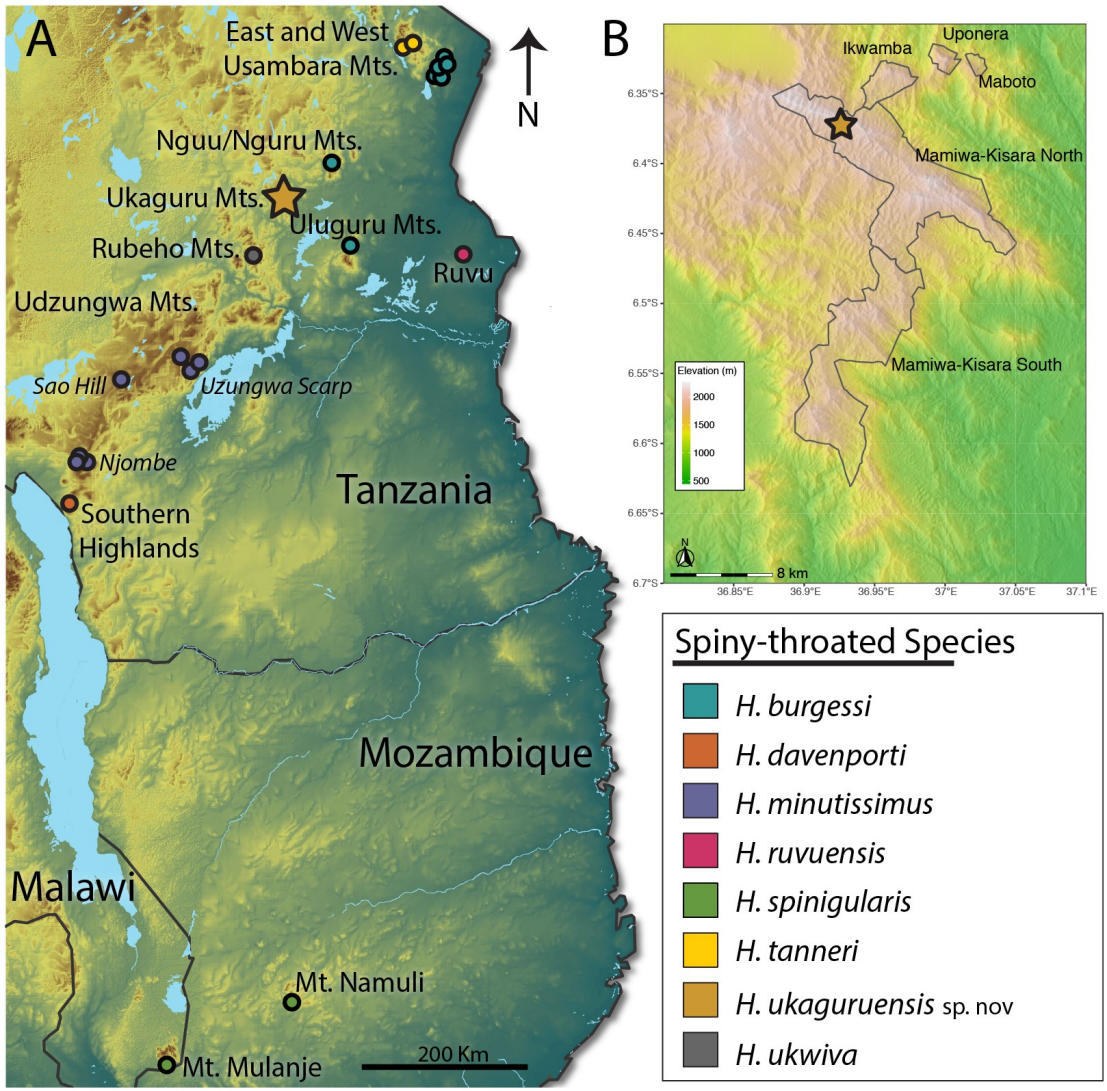
Maps of species sampling and the Ukaguru Mountains forested area. A. Map of the Eastern Afromontane region showing the sampling localities of each member of the spiny-throated reed frog complex included in this study. Elevation is displayed from green colors near sea level and browns for higher elevation areas in the mountains (elevational range is 0–6,000 m above sea level). Mountain blocks and highland areas with spiny-throated reed frog populations are labelled. The locality of the new lineage described here, *H*. *ukaguruensis* sp. nov., is marked by a gold star, while all others are indicated by circles. All colors associated with each species correspond to the color scheme used in all figures. B: Elevational map and outlines of the Ukaguru Forest Reserves with locality of *H*. *ukaguruensis* sp. nov. marked with a gold star.

The East African spiny-throated reed frog complex is comprised of morphologically similar species of small green-brown reed frogs, the males of most of which have small spines on their gular patches, occupying primarily montane forests and grasslands across the Eastern Afromontane region in Tanzania, Malawi, and Mozambique [1–4]. Seven species are currently recognized, (*Hyperolius burgessi*, *H*. *davenporti*, *H*. *minutissimus*, *H*. *ruvuensis*, *H*. *spinigularis*, *H*. *tanneri*, *H*. *ukwiva*), but due to the small, mostly montane-restricted ranges typical of these species (IUCN Red List [www.iucnredlist.org]; Fig 1), and the number of under-surveyed forests in Eastern Africa, diversity in this group is likely underestimated. The morphological similarity of species within this group, and the lack of advertisement calls [4, 5] has led to confusion in identifying and designating new allopatric populations when discovered. However, the combination of molecular divergences, geographic isolation, allopatric distributions, and distinct patterns of gular spines on males has allowed a number of new species to be described recently (most recent update to this clade, *H*. *ruvuensis* [1]).

As currently recognized, *Hyperolius spinigularis* was described by Stevens [4] based on material collected from the base of Mount Mulanje in southern Malawi. Schiøtz [5] originally reported this species also in the East Usambara Mountains in northern Tanzania, however these Northern Tanzanian populations have now been designated as *H*. *burgessi* in the East Usambara, Nguru, and Uluguru mountains [3]. *Hyperolius spinigularis* was also ascribed to populations in the Udzungwa Mountains in Southern Tanzania [4, 7], along with another member of this complex, *H*. *minutissimus* [6]. Recent studies have shown that *H*. *spinigularis* is not found within the Udzungwa Mountains, though two populations of *H*. *minutissumus* appear to have allopatric distributions within the mountain block [3]. *Hyperolius tanneri* is found only within the West Usambara Mountains of northern Tanzania [8]. The most recent discoveries of new species within this radiation were the result of expeditions to new localities where isolated populations were found: *Hyperolius ukwiva* from the Rubeho Mountains in central Tanzania [3], *H*. *davenporti* from the Livingstone Mountains of Tanzania near the Malawian border [3], and *H*. *ruvuensis* from a Tanzanian coastal lowland forest [1].

A recent survey in the Ukaguru Mountains of central Tanzania encountered a phenotypically distinct (golden coloration) spiny-throated reed frog, prompting investigation into the phylogenetic relationships and morphological distinction of this potentially new lineage. As two lineages of this group (*H*. *ukwiva* and *H*. *ruvuensis*), have only been observed once (despite attempts to re-survey) and likely to be highly threatened, new spiny-throated reed frog lineages could be of conservation interest. We investigate morphological, coloration, and genetic differences along with spatial distributions of this lineage compared to all existing lineages to describe a new species within the spiny-throated reed frog complex.

## Methods

### Data compilation

In addition to the newly collected specimens (outlined in the sections below), we compiled relevant information for morphological, genetic and genomic comparative analyses across species of this group. Summary information on localities, and sample sizes for morphology and genetics shown in Table 1.

**Table 1:** Summary information on sample sizes (number of individuals) for morphological and molecular datasets for each lineage. The genetic dataset represents the number of individuals with contributed loci for analyses (16S, RAG1, POMC), with values for each in that order. Some individuals do not have available genetic information for all three loci included. All species except *H*. *ruvuensis* and *H*. *ukwiva* have genomic (ddRAD SNPs). New data generated in this study are all genomic data and new sequences for all genetic loci of two Sao Hill *H*. *minutissimus* and two *H*. *ukaguruensis* sp. nov. (indicated with asterixis). Breakdown of males and females for measurements are shown in Table 3.

Species	Localities	morphological (n)	genetic (n)	genomic (n)
*H*. *burgessi*	3 (Uluguru, Nguru/Nguu, East Usambara)	69	3/10/21	2*
*H*. *davenporti*	1 (Southern Highlands)	14	3/1/3	1*
*H*. *minutissimus*	2 (Uzungwa Scarp, Sao Hill)	13	9/8/7 (2*)	4*
*H*. *ruvuensis*	1 (Ruvu)	4	2/0/0	NA
*H*. *spinigularis*	2 (Mt. Mulanji and Mt. Namuli)	12	2/8/8	2*
*H*. *tanneri*	1 (West Usambara)	4	3/3/3	1*
*H*. *ukaguruensis sp*. *nov*.	1 (Ukaguru)	16*	2/1/2 (2*)	2*
*H*. *ukwiva*	1 (Rubeho)	2	1/1/1	NA

### New field collection

The Mamiwa-Kisara North Forest Reserve in the Ukaguru Mountains was sampled over a seven-night period starting on the 18^th^ of February 2019 for a total of approximately 200 person-hours (Fig 1) [9]. Rains had begun in the region, but the typical heavy spring rains had not yet started for the year. Frogs were caught by hand in small streams and ponds (-6.373, 36.927) on plants above the water (~1–1.5 meters above the water). As found in other species in this clade, no males were calling and all individuals were found through visual searches with flashlights.

*Hyperolius minutissimus* frogs were also collected from a new locality at Sao Hill Forest Plantation (-8.453, 35.170) between 10-12^th^ of February 2019. Four individuals were encountered at the edge of a large wetland on the main road outside of the pine plantation. Searching took place for ~20 person hours at this site over the three nights.

Specimens collected were euthanized using an overdose of benzocaine and fixed in 5% formalin, and subsequently stored in 70% ethanol. Prior to fixing, samples of muscle and/or liver were taken from representative individuals and preserved in 95% ethanol, these specimens are listed in S1 File.

All specimens for this study were collected in accordance with animal ethics guidelines established in the institutions of authors (including the University of Cincinnati (IACUC 21-04-21-02 (LPL), the Natural History Museum London, and approved by the Tanzania Wildlife Research Institute (TAWIRI)).

### Genetics

Total DNA was extracted from tissue preserved in 95% EtOH from one male and one female specimen of the newly identified spiny-throated reed frog from the Ukaguru Mountains and of the Sao Hill *Hyperolius minutissimus* population (n = 4 new extractions) using the DNeasy blood and tissue kit (Qiagen, Valencia, CA). Both male and female specimens were assessed to confirm they were conspecific. This is necessary as males and females are sexually dimorphic, requiring genetic confirmation of conspecific status. All other DNA used in this study was previously extracted using these methods and sequenced following the methods below and downloaded from NCBI GenBank [2].

Extraction, amplification and sequencing followed standard protocols [10]. Each of the newly collected individuals was DNA barcoded to verify its identity using the mitochondrial 16S rRNA locus (483 bp). These individuals were also sequenced for the nuclear genes POMC (Pro-opiomelanocortin: exon 629 bp) and RAG-1 (Recombination activating gene: exon 782 bp) to complement existing genetic datasets of this clade [11]. Primers and PCR conditions of POMC and RAG-1 are the same as in [2, 11]. Primers for 16S were 16SC and 16SD [12]. Amplification followed standard PCR conditions [13] with the following thermal cycle profile: 2 min at 94°C, followed by 35 cycles of 94°C for 30 sec, 46°C for 30 sec, 72°C for 60 sec and a final extension phase at 72°C for 7 min. All amplified PCR products were verified using electrophoresis on a 1.0% agarose gel stained with SYBR Safe DNA gel stain (Invitrogen Corporation, Carlsbad, CA, USA). PCR products were purified using the Qiagen DNeasy DNA Purification System according to the manufacturer’s recommendations. DNA sequences were obtained on an ABI PRISM 3730xl DNA sequencer. Editing and assembly of contigs were completed in Geneious Prime 2019.2.1 (https://www.geneious.com). See S1 File for the complete dataset of sanger sequences and GenBank accession numbers.

Phylogenetic relationships and structure within this clade were assessed in two ways. The mitochondrial data (16S), which had high levels of sequence divergence was assessed using phylogenetic tree methods (outlined below). Nuclear genes, which had much lower levels of divergence and may still retain strong signals of incomplete lineage sorting, were assessed as haplotype networks to reveal structure without enforcing unrealistic tree structure for slow evolving genes. A species tree using the StarBeast function in BEAST was also created combining nuclear and mitochondrial loci (see below).

16S mtDNA sequences for all known members of the spiny-throated reed frog clade were obtained from GenBank and incorporated in the mitochondrial gene tree analysis along with the four newly-sequenced specimens (male and female of the new species and a male and female *H*. *minutissimus* from the Sao Hill locality). The resulting sequences were aligned in MUSCLE v3.7 [14] in the CIPRES gateway server [15] with default settings.

The evolutionary relationships of the species based on the 16S barcode mtDNA alignment were reconstructed using Bayesian inference (MrBayes v3.2; [16] and BEAST2 v2.5.2; [17]) and maximum likelihood (RAxML v8.2.12; [18]). MrBayes and RAxML were also run on the CIPRES server while BEAST2 was run on a local installation. Reconstructions were performed both with an outgroup (*H*. *mitchelli*–spiny-throated reed frogs are monophyletic and sister to a large clade of *Hyperolius* including *H*. *mitchelli* [19]) and without (midbranch rooting in BEAST). The topology did not change between these methods. The substitution model used for 16S was HKY+I+G, selected using jModelTest2 [20, 21] on the CIPRES server, based on Akaike Information Criterion (AIC and AICc).

In the MrBayes analyses, two runs with four Markov chains were run for 10 million generations and sampled every 1000 generations with heating parameter of 0.2; discarding the first 25% as burn-in. RAxML was run allowing the bootstrapping to halt automatically under default settings for RAxML-HPC BlackBox. In BEAST2, a relaxed lognormal clock was used to accommodate some potential variation of rates within the group. A Yule tree prior was used with a uniform birth rate, exponential gamma shape, log normal HKY transition/transversion (as identified by jModelTest2), and uniform prior for proportion invariant sites. Other models (Birth-Death) gave identical topologies, but without an explicit expectation of extinctions, the Yule model results are shown. The run consisted of 15 million generations logging every 1000 with the first 10% discarded as burn-in. An assessment of model performance was completed in Tracer v1.7.1 [22] for BEAST2, with ESS values above 200 and visual inspection of mixing. A final tree was constructed using TreeAnnotator v 2.6.0 in BEAST2 and viewed in FigTree v1.4.0 (http://tree.bio.ed.ac.uk/software/figtree/).

Pairwise population divergence and within population divergence was calculated using MEGA7 [23]. Within-group average distance (p) used uniform rates, pairwise deletion for missing data, and 500 bootstraps.

Nuclear loci (POMC and RAG-1) were also downloaded from GenBank and combined with newly sequenced individuals. All loci aligned unambiguously. Divergence in nuclear genes were visualized as unrooted TCS allele networks [24] using PopART v1 [25].

A “species tree” in StarBeast combining datasets (19 samples across all species with all gene represented except *H*. *ruvuensis* which only has 16S sequences) was also evaluated. A relative mitochondrial clock rate was set at 5.0, with nuclear clock rates of 1.0 to reflect estimates of relative rates [26] with “ploidy” also specified for the multispecies coalescent ("Y or mitochondrial" vs "autosomal_nuclear"). Due to difficulties in convergence as assessed through Tracer [27], strict molecular clocks were used for all genes. The species tree model was set to Yule, and analyses were run for 20 million generations sampling every 5000, with 10% burn-in.

### Genomics

A smaller dataset (12 individuals) was used to create a reduced-representation whole-genome single nucleotide polymorphism (SNP) dataset through double-digest RAD-sequencing (ddRAD-seq) (S1 and S2 Files). One individual per species per locality was used except where noted (Table 1). Crucially, DNA extracted from *Hyperolius ukwiva* and *H*. *ruvuensis* was not sufficient in quantity and/or quality for ddRAD-seq. We are not aware of the existence of further tissues in museum collections. Samples were extracted from fresh tissues (DNEasy kit) or from existing, high-quality DNA. The libraries were quantified using a Qubit fluorometer (Life Technologies, USA), normalized to 500 ng DNA when possible (>100 ng minimum), and sent to Admira Genomics (Genohub project, USA) for sequencing. The ddRAD-seq library was prepared with EcoRI-MspI restriction enzymes and Illumina HiSeq 2500 150 × 2 pair-ended sequencing.

The STACKS pipeline v2.60 [28–30] was used to identify SNPs. STACKS parameters were explored through the R80 method [31] and estimates comparing values 2 through 7 were explored for M = n for *de novo* ddRAD-seq assembly and SNP discovery, as well as downstream analyses. As only 1–2 individuals were representative of each population, all individuals were listed as a single population for the R80 method (STACKS author’s recommendation, J. Catchen). M & n = 4 were ultimately used to balance the diversity of samples within the dataset and identifying true SNPs. In total, 821,683 loci were generated, composed of 214,643,861 sites and 2,201,442 variant sites. Due to the need for a very complete dataset, only loci present in every population (-p 10) were retained. This yielded a total of 1,909 loci. The mean effective per-sample coverage was 18.3x (stdev = 2.8x, min = 11.4x, max = 22.3x). The mean number of sites per locus was 257.5. A consistent phasing was found for 95.2% of diploid loci needing phasing.

Finally, only the first SNP of each locus was included in the final dataset to create unlinked loci, crucial for SNAPP (SNP and AFLP Package for Phylogenetic analysis, [32]) and STRUCTURE [33] analyses in this non-referenced dataset (see below). This dataset was also used for concatenated phylogenetic tree construction using BEAST2, MrBayes, and RAxML. A non-concatenated tree was created through a SNAPP analysis. jModelTest was used to determine the best nucleotide substitution model for the SNP dataset. Our best fit model, selected through AIC, was GTR.

MrBayes and RAxML analyses were run on the CIPRES server (https://www.phylo.org) with the GTR site substitution model. MrBayes v 3.2.7 was run for 500,000 MCMC steps, with a burn-in of 25%, and sampling occurred every 500 iterations. Effective chain mixing and effective sample sizes (ESS) across parameters (≥ 100) were assessed in Tracer (v1.7.1). BEAST2 (v. 2.6.0) analyses were conducted in two ways, as a concatenated SNP tree (matching MrBayes methodology) and as a SNAPP tree [32], both using a relaxed lognormal molecular clock. SNAPP (v 1.5.2) analyses use bi-allelic SNPs to derive a posterior distribution of putative species trees through estimating the probability of allele frequency changes across nodes given the data. Both concatenated BEAST and SNAPP analyses were run for 100,000,000 iterations, with 10% burn-in, and sampling every 1,000 iterations. To ensure that the effective sample sizes (ESS) across parameters were ≥ 100, all results were assessed in Tracer v 1.7.1. A final concatenated trees was constructed using TreeAnnotator v 2.6.0 and viewed in FigTree v1.4.0, while the SNAPP tree was viewed in Densitree v 2.2.7 (https://www.cs.auckland.ac.nz/~remco/DensiTree/). RAxML v 8.2.12 was run using the GTR-GAMMA model of sequence evolution and automatic bootstrapping.

STRUCTURE (v 2.3.4) was used for population genetic analyses on the phased SNP data. Population clusters (K) between 1–12 were assessed, with 10 iterations each. The burn-in period was 100,000, and the MCMC reps were set at 200,000. We used the admixture model without a population prior. All other parameters were set to default values. The program Structure Harvester v 0.6.94 [34] was used to implement the Evanno et al. *ad hoc* method of K estimation [35], which detects the uppermost level of hierarchy.

### Morphology

Individuals of the new species were all collected from the same locality (-6.37272, 36.92722, 1862m elevation) in Mamiwa-Kisara North Forest Reserve on the Ukaguru Mountains of Tanzania. Adult (>20mm) specimens of both sexes were measured for 17 standard morphological traits (See Fig 2 of Greenwood et al. 2020 [36] for visualization of measurements). All adult males had gular sacs, and many females of this size were gravid. Juveniles were significantly smaller at the time of collection. These complement the existing morphological dataset for spiny-throated reed frogs [1, 3]. Measurements taken were: Snout-Urostyle Length (SUL), Head Width (HW), Head Length Diagonal from corner of mouth (HLD), Head Length Diagonal from jawbone end (HLDJ), Nostril-Snout (NS), Inter-narial (IN), Eye to Nostril (EN), Eye Distance (EE), Inter-orbital (IO), Tibiofibula Length as approximated by measuring the crus (TL), Thigh Length (THL), Tibiale Fibulare Length as approximated by measuring the tarsus (TFL), Foot Length (FL), Forelimb Length (FLL), Hand Length (HL), Width of Gular Flap (WGF), Height of Gular Flap (HGF). Measurements were taken with Mitutoyo CD-6 electronic calipers by a single investigator (Loader) to reduce variation in measurements and preserve continuity across all previous measurements within the clade. Measurements were taken to the nearest 0.1 mm. The final dataset consisted of 12 males and three females with sex being determined by the presence (male) or absence (female) of the gular flap. Qualitative gular characters in males (shape, relative proportions, and ‘spinosity’), which had previously proved useful for species designation in this group [3] were investigated as well.

The spiny-throated reed frogs are a very morphologically distinct monophyletic group with characteristic body proportions of these small bodied frogs with diagnostic gular spines on males of all species except *H*. *tanneri* [3], thus comparisons of morphometrics were only made to the other members of this species complex (following initial DNA analyses confirming membership). Measurements of the seven other members of this complex currently described (*H*. *burgessi*, *H*. *davenporti*, *H*. *minutissimus*, *H*. *spinigularis*, *H*. *tanneri*, *H*. *ruvuensis*, and *H*. *ukwiva*) were taken from [1, 3] and were measured by the same researcher (Loader) across all datasets. Only two female *H*. *ukwiva* were available, but photographs of a male taken in the field allow characterization of the gular spines (see species description in [3].

Morphological differences between lineages were assessed by exploring overall morphology distinctiveness using a Principal Component analysis (PCA) on log-transformed measurements. As females tend to be larger than males, do not have gular flaps and are represented by lower sample sizes, this PCA was restricted to male specimens only. Morphological traits identified as being potential diagnostic features distinguishing the new species from the rest of the species group were explored further by means of statistical hypothesis testing for both males and females separately. These features were: overall body size (general loading of PC1), eye size (most important loading of PC2) and gular flap width (most important loading of PC3). Differences in SUL, eye diameter/head width ratio and gular flap width/height ratio across species were therefore tested using univariate ANOVAs and post-hoc Tukey tests. All analyses were performed in R v 3.6.1 [37] on log10 transformed measurements.

### Nomenclatural acts

The electronic edition of this article conforms to the requirements of the amended International Code of Zoological Nomenclature, and hence the new names contained herein are available under that Code from the electronic edition of this article. This published work and the nomenclatural acts it contains have been registered in ZooBank, the online registration system for the ICZN. The ZooBank LSIDs (Life Science Identifiers) can be resolved and the associated information viewed through any standard web browser by appending the LSID to the prefix "http://zoobank.org/". The LSID for this publication is: urn:lsid:zoobank.org:pub: A2AA3A2D-29F8-46A6-A328-88E8C280E110. The electronic edition of this work was published in a journal with an ISSN, and has been archived and is available from the following digital repositories: PubMed Central and LOCKSS.

## Results

### Phylogenetics

**Fig 2:**
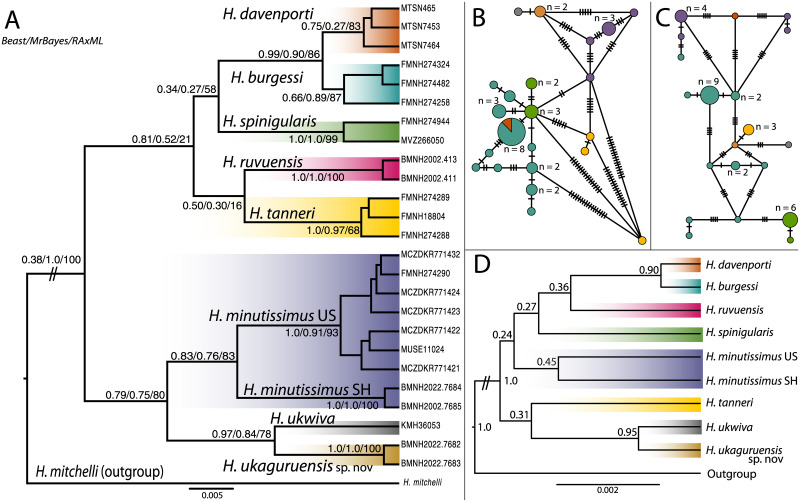
Phylogenetic relationships between members of the spiny-throated reed frog complex from both mitochondrial and nuclear markers. The color scheme corresponding to each species is indicated on the phylogenetic tree and corresponds to all figures in the manuscript. A. Phylogenetic tree of specimens from the spiny-throated reed frog complex based on 16S mitochondrial data. Museum IDs or field IDs are listed for each individual included. Maximum clade credibility tree from BEAST2 shown, with branch lengths corresponding to sequence divergence. Values on nodes reflect posterior and bootstrap estimations from BEAST2/MrBayes/RAxML respectively. While the overall tree is mostly stable across estimation methods, the placement of *Hyperolius tanneri* is uncertain between methods, indicated by “-”and ** to show that this relationship is only supported in BEAST. B. TCS haplotype network based on the nuclear POMC alignment. Each circle node represents a unique haplotype and the size is proportional to the number of samples it represents. Only one haplotype has more than one species that share it, where one *H*. *davenporti* has a common *H*. *burgessi* haplotype. Species are indicated in color, and hypothesized intermediates are shown as solid circles. Crossed lines on the network indicate single nucleotide polymorphisms. C. TCS haplotype network based on the nuclear RAG1 alignment. All characteristics are shared with panel B. No haplotypes are shared between species and circles are not proportional to panel B. D. StarBeast species tree. All species are represented by at least one individual with all loci sequenced except *H*. *ruvuensis* which only has 16S data available.

Based on 16S mitochondrial sequences, all previously recognized species and the new species (*H*. *ukaguruensis* sp. nov.) represent monophyletic lineages (Fig 2a). Deep sequence divergence within *H*. *minutissimus* was also discovered, corresponding with distinct collection localities in the Udzungwa Mountain Block: region of the Uzungwa Scarp and the Sao Hill area with approximately 80 km separating localities (see below). As the type locality for *H*. *minutissimus* (Southern Highlands) was not sampled for genetics or morphology, and no morphological measurements were taken of the Sao Hill *H*. *minutissimus* specimens, we refrain from assigning either lineage a new name. We nonetheless treat them as distinct phylogenetic lineages from here on out, referring to these by their localities (Uzungwa Scarp = US, Sao Hill = SH). All morphological measurements however, correspond to the Uzungwa Scarp lineage.

Phylogenetic tree reconstructions based on 16S confirmed that all individuals clustered as separate species, indicating that species tree analyses would not violate the assumption of lack of mixing across lineages. Deeper within the tree than the species level, however, confidence across nodes was only moderate and various methods yielded conflicting results (Fig 2A). The main uncertainty concerned the placement of *H*. *tanneri*. BEAST2 and MrBayes analyses placed it in a clade with *H*. *spinigularis*, *H*. *ruvuensis*, *H*. *burgessi*, and *H*. *davenporti*, though MrBayes showed an unresolved polytomy and BEAST2 assigned it as sister to *H*. *ruvuensis* with weak support. RAxML identified a basal polytomy with weak support (Fig 2A).

Most recent nodes had better posterior and bootstrap support. The new species described here (*H*. *ukaguruensis* sp. nov.) has support as the sister to the poorly known *H*. *ukwiva* from the Rubeho Mountains (Fig 1). This relationship is supported in nuclear genes as well. In POMC, the single *H*. *ukwiva* individual is only one base pair diverged from the two *H*. *ukaguruensis* sp. nov. individuals (Fig 2B). In the RAG-1 haplotype network, there also is a direct connection between these two lineages (three base pairs) (Fig 2C). Haplotype networks of both nuclear genes showed substantial variation in *H*. *burgessi* individuals and *H*. *minutissimus*. The StarBeast species tree had low node support despite long run times (Fig 2D), but showed the same structure as genomic analyses (below).

Mitochondrial distances for 16S show 2.9% species divergence between *H*. *ukaguruensis* sp. nov. and its nearest relative, *H*. *ukwiva* (Table 2).

**Table 2:** Genetic distance (patristic) between species based on 16s sequence data. *Hyperolius minutissimus* is broken down into two separate lineages based on locality (Sao Hill = SH, Uzungwa Scarp = US) due to significant structure. Standard error from 100 bootstrap replicates is shown in parentheses for each pairwise distance.

	*H*. *minutissimus SH*	*H*. *minutissimus US*	*H*. *davenporti*	*H*. *burgessi*	*H*. *spinigularis*	*H*. *ruvuensis*	*H*. *tanneri*	*H*. *ukwiva*	*H*. *ukaguruensis*
*H*. *minutissimus SH*	-								
*H*. *minutissimus US*	3.3% (0.8%)	-							
*H*. *davenporti*	4% (0.9%)	4.6% (0.9%)	-						
*H*. *burgessi*	4.3% (0.9%)	4.9% (0.9%)	0.6% (0.3%)	-					
*H*. *spinigularis*	5% (1.0%)	5.6% (1.0%)	2.6% (0.7%)	2.8% (0.7%)	-				
*H*. *ruvuensis*	3.6% (0.8%)	5.3% (0.9%)	1.5% (0.5%)	1.9% (0.6%)	3.2% (0.8%)	-			
*H*. *tanneri*	4% (0.8%)	4.2% (0.9%)	2.4% (0.6%)	2.7% (0.7%)	3.6% (0.7%)	2.0% (0.6%)	-		
*H*. *ukwiva*	4.3% (0.9%)	5% (1.0%)	4.4% (1.0%)	4.7% (1.0%)	5.2% (1.0%)	4.8% (1.0%)	5.0% (1.0%)	-	
*H*. *ukaguruensis*	3.5% (0.8%)	3.8% (0.8%)	3.3% (0.8%)	3.7% (0.8%)	4.6% (0.9%)	4.2% (0.9%)	4.3% (0.9%)	2.9% (0.7%)	-

### Genomics

**Fig 3:**
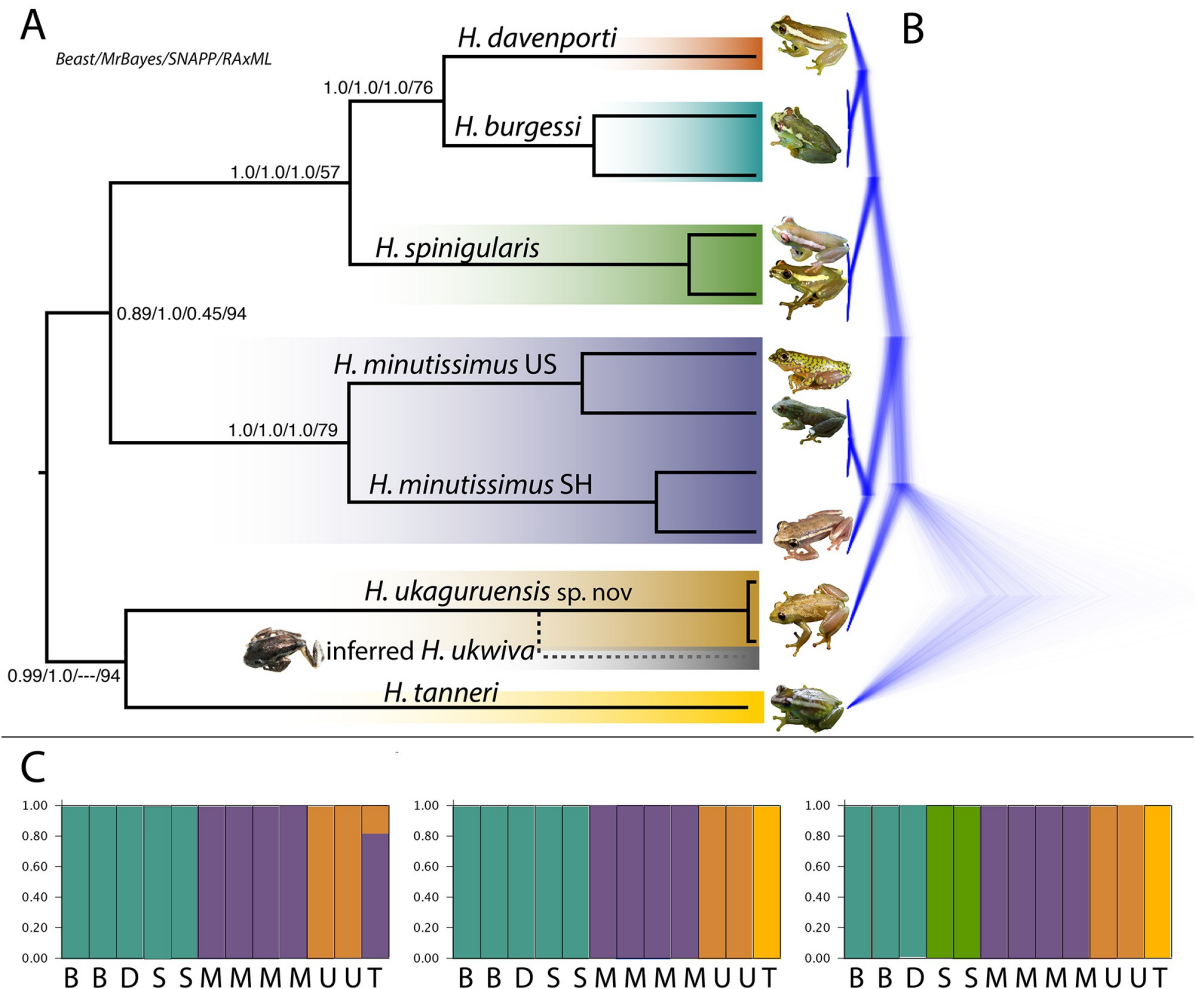
Genomic comparisons between the members of the spiny-throated reed frogs. A. Maximum clade credibility tree from BEAST2 shown, with branch lengths corresponding to sequence divergence. Values on nodes reflect posterior and bootstrap estimations from BEAST2, MrBayes, SNAPP, and RAxML respectively. Photos of each clade are shown. *Hyperolius ukwiva* is included as “inferred” due to its close mitochondrial and nuclear affinities in the genetic dataset to *H*. *ukaguruensis* sp. nov. and the results of the species tree (16S, RAG-1, POMC analysis). B. SNAPP tree showing gene trees from the top supported phylogeny (“blue trees”). This tree differs in whether *H*. *tanneri* is basal to all or in a basal sister lineage with *H*. *ukaguruensis*. C. STRUCTURE analysis showing K 3–5. Each species is indicated by the first letter of their name (e.g., *H*. *burgessi*–B, *H*. *spinigularis* = S, etc.).

After filtering the “first SNP per locus” dataset to only SNPs found in every population, 1909 SNPs remained for genomic analyses (S2 File). The best fitting substitution model identified for the SNP dataset through both AIC and AICc was GTR. Phylogenomic and population genomic analyses had overall high support for 3–5 major groupings (Fig 3). This included a “spinigularis” clade (*H*. *davenporti*, *H*. *burgessi*, *H*. *spinigularis*), a “minutissimus” clade (*H*. *minutissimus* populations from two different areas in the Udzungwa Mountains), *H*. *tanneri*, and *H*. *ukaguruensis* sp. nov. The SNAPP analysis predicted the placement of *H*. *tanneri* as basal, with *H*. *ukaguruensis* sp. nov. as the next diverging lineage (Fig 3b), while the other methods found *H*. *tanneri* and *H*. *ukaguruensis* sp. nov. to be sister to each other, though on a very deep branch.

STRUCTURE analyses can be difficult to interpret in a framework with only one to two individuals per species, but the grouping of individuals in K 3–5 show some aspects of affinity which support the distinctness of the “spinigularis”, “minutissimus”, *H*. *tanneri* and *H*. *ukaguruensis* sp. nov. lineages. While K = 3 was selected from the Evanno method, K = 4 and 5 were also significant gains in likelihood with clear population assignments and are included for reference.

### Morphology

**Fig 4:**
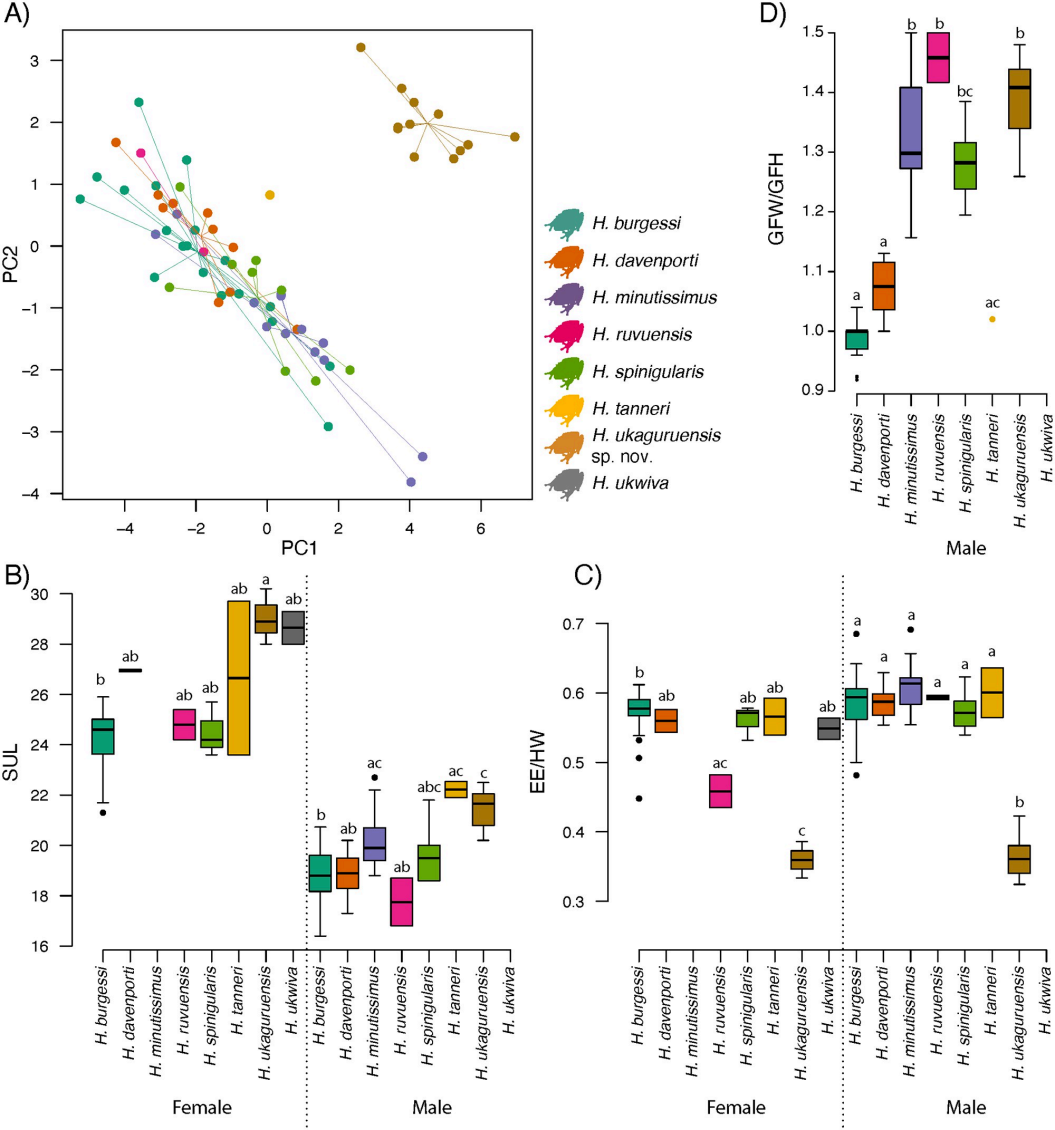
Morphological comparison of spiny-throated reed frogs. A) PCA of all morphological variables comparing males of eight species. B) Snout-urosyle Length for females and males. C) Eye diameter/head width ratios for females and males. D) Gular flap width/height ratios for males. Letters above boxplots indicate post-hoc groups at p<0.001. The only known male specimen of *H*. *ukwiva* could not be located for measurements (only casual photos were available), thus measurements of this individual were not able to be included.

Summary statistics for all linear morphological measurements per species are presented in Table 3 (and all measurements provided as S3 File). The PCA performed on male specimens shows that the new species, *H*. *ukaguruensis* sp. nov., represents a clear outlier when plotting the first two principal components (62.9% cumulative variance, Fig 4a). These axes represent overall size differences (no single main variable contributing to PC1) and eye diameter (variable most contributing to PC2; S4 File). Gular flap height was the variable contributing most to PC3 (S3 File).

**Table 3:** Mean and standard deviation of all measurements for H. spinigularis species groups. Most data was taken from Lawson et al. (2015) and Barrat et al. (2017) with the addition of H. ukaguruensis data from this paper.

species	sex	N	SUL	HW	HLD	HLDJ	NS	IN	EN	EE	IO
H. burgessi	f	28	24.27±1.24	8.47±0.53	7.19±0.56	8.26±0.70	1.25±0.13	2.36±0.14	2.27±0.27	4.83±0.30	3.14±0.31
	m	41	18.77±1.10	6.51±0.39	5.78±0.36	6.60±0.40	0.99±0.12	1.89±0.14	1.77±0.20	3.82±0.36	2.52±0.20
H. davenporti	f	2	26.95±0.07	9.20±0.00	6.90±0.00	8.40±0.28	1.25±0.07	2.25±0.07	2.60±0.14	5.15±0.21	2.80±0.14
	m	11	18.82±0.92	6.47±0.28	5.45±0.32	6.33±0.36	1.04±0.08	1.91±0.12	1.87±0.09	3.79±0.19	2.42±0.16
H. minutissimus	f	0									
	m	13	20.32±1.17	6.83±0.53	6.06±0.42	6.50±0.00	1.03±0.08	1.96±0.12	2.00±0.15	4.17±0.43	2.41±0.22
H. ruvuensis	f	2	24.8±0.85	8.75±0.64	7.05±0.21	8.2±0.14	1.25±0.07	2.4±0.14	2.25±0.07	4±0	4.65±0.21
	m	2	17.75±1.34	6.15±0.07	5.35±0.07	6.35±0.07	1.05±0.07	2.1±0	1.9±0	3.65±0.07	2.9±0.28
H. spinigularis	f	3	24.50±1.08	8.70±0.61	7.33±0.45	8.77±0.64	1.30±0.00	2.33±0.06	2.60±0.10	4.87±0.12	3.07±0.31
	m	9	19.59±1.02	6.87±0.41	6.08±0.40	6.88±0.39	1.13±0.07	1.90±0.13	1.96±0.18	3.93±0.20	2.48±0.26
H. tanneri	f	2	26.65±4.31	9.15±1.48	7.60±1.13	8.70±0.71	1.50±0.28	2.35±0.49	2.65±0.21	5.15±0.49	2.90±0.42
	m	2	22.22±0.45	6.83±0.88	6.08±0.53	6.86±0.51	1.16±0.06	1.96±0.22	2.12±0.26	4.12±0.88	2.68±0.40
H. ukaguruensis	f	3	29.03±1.11	9.80±0.70	7.67±0.25	8.57±0.67	1.77±0.21	2.37±0.42	2.07±0.25	3.53±0.47	5.20±0.20
	m	12	21.44±0.79	7.19±0.25	6.47±0.34	6.87±0.19	1.67±0.21	2.22±0.26	1.96±0.16	2.62±0.20	4.02±0.22
H. ukwiva	f	2	28.65±0.92	9.95±0.78	8.00±0.14	9.80±0.71	1.45±0.21	2.75±0.35	2.70±0.14	5.45±0.21	3.20±0.14
	m										
species	sex	N	TL	THL	TFL	FL	FLL	HL	GFW	GFH	
H. burgessi	f	28	12.06±0.78	11.23±0.92	7.64±0.39	10.34±0.72	5.35±0.31	7.12±0.43			
	m	41	9.12±0.46	8.43±0.62	5.84±0.27	8.04±0.61	4.21±0.34	5.38±0.52	5.01±0.44	5.09±0.44	
H. davenporti	f	2	12.85±0.35	12.95±0.64	8.55±0.07	10.7±0.28	6.3±0.71	7.35±0.21			
	m	11	9±0.50	8.96±0.50	5.72±0.42	7.44±0.65	4.28±0.30	5.36±0.32	5.35±0.34	5.00±0.38	
H. minutissimus	f	0									
	m	13	9.88±0.55	9.52±0.62	6.19±0.54	8.81±0.75	4.69±0.38	5.83±0.44	5.93±0.42	4.46±0.37	
H. ruvuensis	f	2	12.05±0.35	11.6±0.28	7.2±0.14	10.2±0.14	5.25±0.07	6.65±0.07			
	m	2	8.9±0.28	8.85±0.49	5.55±0.35	7.5±0.14	4.5±0.42	4.85±0.07	4.95±0.21	3.4±0.28	
H. spinigularis	f	3	12.93±0.32	11.83±0.59	8.2±0.46	11.13±0.83	5.63±0.35	7.03±0.38			
	m	9	9.87±0.34	8.83±0.63	6.21±0.43	8.24±0.67	4.47±0.24	5.27±0.37	5.09±0.36	3.96±0.28	
H. tanneri	f	2	14.95±5.73	11.15±3.32	7.7±2.26	10.65±3.04	6.1±1.70	7.35±1.48			
	m	2	11.11±0.69	8.84±1.61	5.94±1.05	9.49±1.11	4.72±0.45	6.32±0.74	5.00±0.31	4.90±0.30	
H. ukaguruensis	f	3	15.1±0.87	13.4±0.35	7.93±0.85	13.6±0.72	6.43±0.32	8.27±0.75			
	m	12	10.47±0.34	9.05±0.78	6.09±0.50	9.73±0.47	4.66±0.34	6.66±0.37	6.99±0.31	5.05±0.30	
H. ukwiva	f	2	14.2±1.13	14.05±0.78	8.85±0.21	12.65±0.49	7.45±0.35	9±0.85			
	m										

There are significant size (SUL) differences among species of the *H*. *spinigularis* group (males: F = 14.52, Df = 6,83, p<0.001; females: F = 7.99, Df = 6,35, p<0.001) with posthoc tests showing that male *H*. *ukaguruensis* sp. nov. are significantly larger in size (p<0.001) than *H*. *burgessi*, *H*. *davenporti*, *H*. *ruvuensis* and *H*. *spinigularis* (note: no male measurements were available for *H*. *ukwiva*) and females significantly larger than *H*. *burgessi* and *H*. *spinigularis* (p<0.001; Fig 4b). It is important to point out however that for all species except *H*. *burgessi*, only 3 or less female specimens were available (Table 3). More striking are the differences in eye size relative to head width (males: F = 74.41, Df = 6,83, p<0.001; females: F = 26.91, Df = 6,35, p<0.001) with *post hoc* tests showing that for males, *H*. *ukaguruensis* sp. nov. has significantly smaller eyes relative to head width than all others in this species group (p<0.001; Fig 4c), and for females, all others but *H*. *ruvuensis*, though here the paired-test post hoc difference could still be considered significant with p = 0.01.

The gular flap shape (males only) was also significantly different between species (F = 74.23, Df = 6,59, p<0.001), with that of *H*. *ukaguruensis* sp. nov. being wider than long, significantly more so than *H*. *burgessi*, *H*. *davenporti*, and *H*. *tanneri* (p<0.001; Fig 4d). To summarize, *H*. *ukaguruensis* sp. nov. is thus a large species of spiny-throated reed frog with comparably small eyes, which are unmistakably smaller than those of other species. In addition, males have gular flaps that are wider than long.

**Fig 5:**
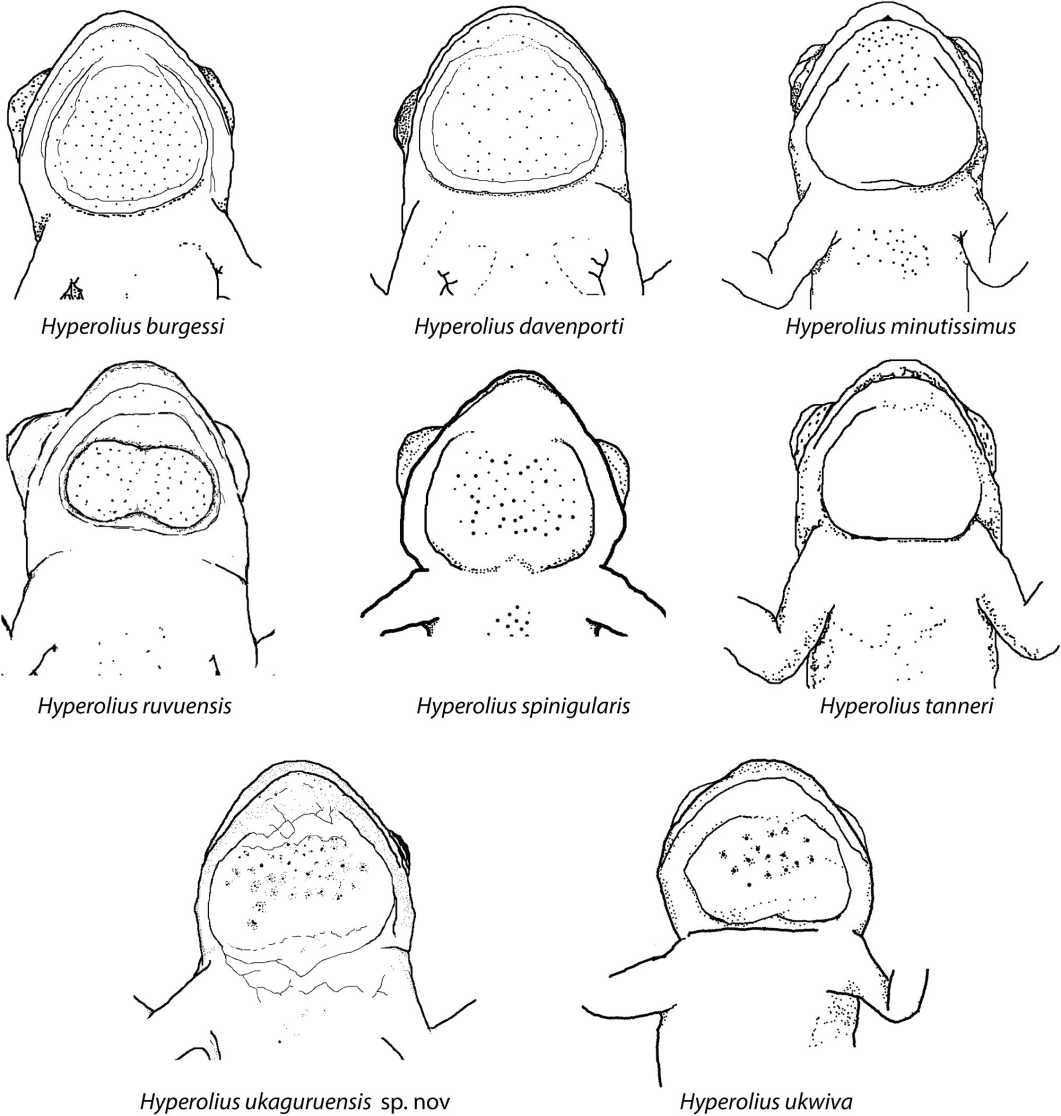
Schematic drawings of the ventral view of head region of *H*. *ukaguruensis* sp. nov. and comparison to previous drawings from Barratt *et al*. (2017) and Loader *et al*. (2015).

Two main groupings of shape emerge, with *H*. *tanneri*, *H*. *davenporti*, and *H*. *burgessi* possessing more circular gular shapes (GFW/GFH ~ 1.0) and *H*. *minutissimus*, *H*. *ruvuensis*, *H*. *spinigularis*, and *H*. *ukaguruensis* sp. nov. possessing wider oval shaped gular flaps (Schematic, Fig 5). Qualitatively, the gular flap of *H*. *ukaguruensis* sp. nov. is similar to the closely related *H*. *ukwiva* for which no formal measurements are available (Figs 2 & 3). Spines are clustered primarily near the top of the gular patch and not extending far into the posterior area of the gular flap with sparse asperities on the chin and body.

### Summary of distinctness

**Fig 6:**
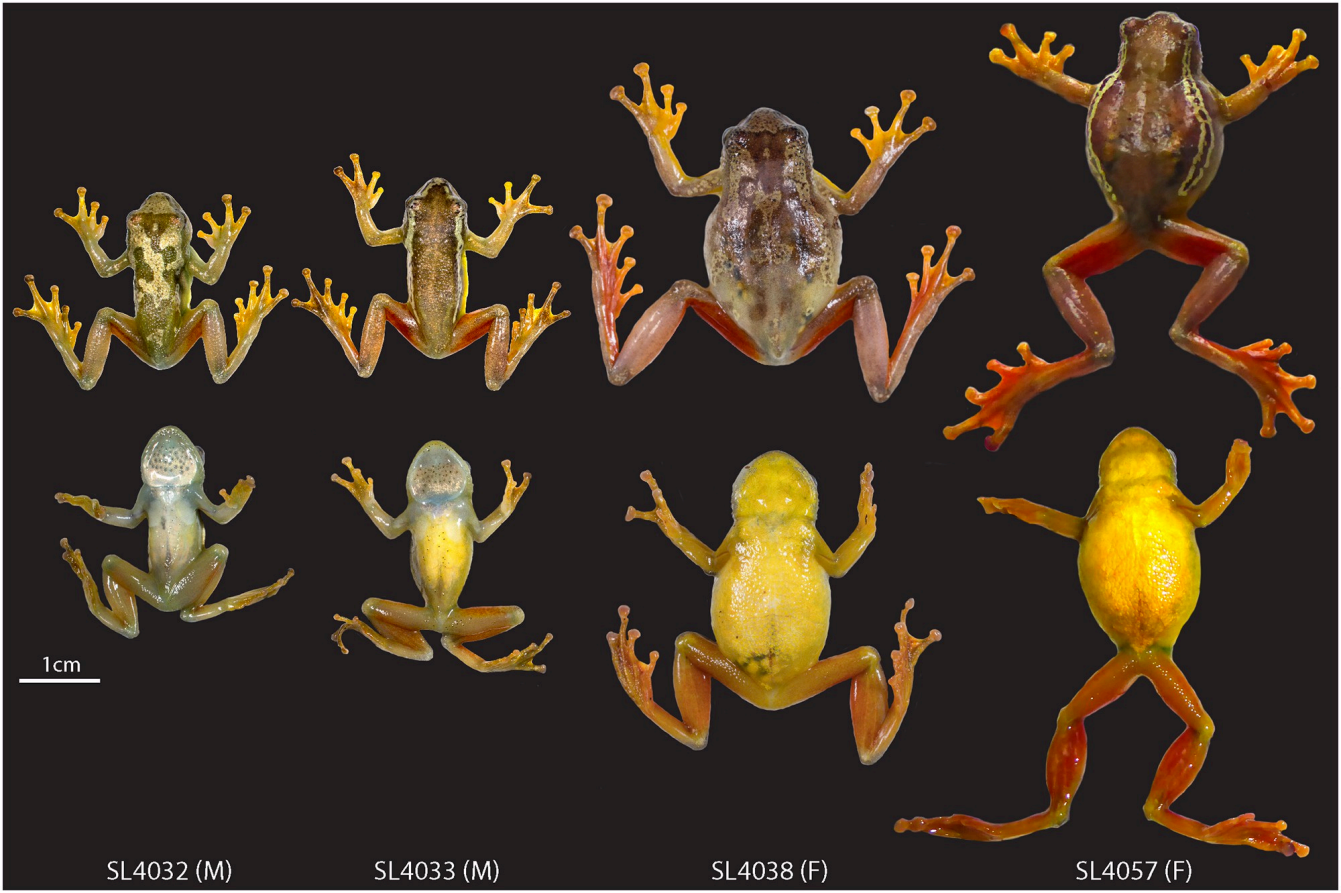
Dorsal and ventral view of anaesthetized male and female type specimens showing dorsal color polymorphism. From left to right: BMNH 2022.7682 (holotype; male), field ID SL_4033 (male), BMNH 2022.7683 (female) and field ID SL-4057 (female). Photos by H. C. Liedtke.

The new lineage discovered here appears to only occur in the Ukaguru Mountains of Tanzania and not in any other mountain blocks within the East African range of the spiny-throated reed frog clade (Fig 1). No other populations have the same distinct coloration which is warmer in hue (“golden”) than the other members which are cooler greens and browns (Fig 6), though *H*. *ukwiva* has “warm brown” coloration. In addition, no other population appears to contain individuals that are genetically the same as these specimens or have similar morphometric measurements (though overall morphological variation is remarkably conserved in *Hyperolius* frogs in general and in the subset of spiny-throated reed frogs).

Most importantly, this lineage is distinct from its closest relative, *H*. *ukwiva*. Genetically it is distinct (2.9% mitochondrial divergence, and unique nuclear haplotypes), and morphologically it is distinct in female eye to head width ratios (no male *H*. *ukwiva* available for comparison).

### Description

***Hyperolius ukaguruensis* sp. nov**. urn:lsid:zoobank.org:act:4166708E-2561-4085-B29A-4FAE83510661

*Holotype*.—BMNH 2022.7682 (male) collected in Mamiwa Kisara Forest Reserve, Ukaguru Mountains, Tanzania (-6.37272, 36.92722, elevation 1862m) on 19 February 2019 by Lawson, Loader, Lyakurwa, Liedtke.

*Paratypes*.—Male: Field numbers SL_4033–4037, 4059–4063. Female: BMNH 2022.7683, Field numbers SL_4057, 4058. All paratypes were collected in the same small stream as holotype but collected between 19^th^– 23^rd^ February.

*Diagnosis*.—Horizontal pupil with distinctive gular flap in males. As with most other members of the spiny-throated group (*H*. *spinigularis*, *H*. *burgessi*, *H*. *davenporti*, *H*. *minutissimus*, *H*. *ruvuensis*, *H*. *ukwiva*), *H*. *ukaguruensis* sp. nov. also has the presence of dermal asperities (including the body and chin region) on the ventrum. This trait is unique amongst members of the genus *Hyperolius*. The presence of asperities on the gular flap diagnoses *H*. *ukaguruensis* sp. nov. from *H*. *tanneri*, for which they are absent. *Hyperolius ukaguruensis* sp. nov. primarily has asperities anteriorly positioned (closer to the mouth), which differentiates it from *H*. *spinigularis*, *H*. *burgessi* and *H*. *davenporti* which have an even distribution of dermal asperities on the gular flap. *Hyperolius minutissimus* and *H*. *ukwiva* have a similar distribution of asperities to *H*. *ukaguruensis* sp. nov.. Furthermore, in males, *H*. *ukaguruensis* sp. nov has a distinctively shaped gular flap, differentiating it from *H*. *davenporti*, *H*. *tanneri* and *H*. *burgessi* (Figs 4 & 5). Quantification of differences in gular shape from the *H*. *ukwiva* were not possible due to inability to locate the only known male specimen, but should be investigated in future studies.

Females are larger in *H*. *ukaguruensis*, *H*. *ukwiva*, and *H*. *tanneri*, reaching sizes >25mm, substantially larger than females of *H*. *minutissimus* (18–24 mm [6], no females available to measure for this analysis) or *H*. *burgessi*, *H*. *davenporti*, *H*. *ruvuensis*, and *H*. *spinigularis* (Fig 4, Table 3). Males of *H*. *ukaguruensis* sp. nov. were significantly bigger than most species (*H*. *burgessi*, *H*. *davenporti*, *H*. *ruvuensis*), and were much bigger on average than *H*. *minutissimus* and *H*. *spinigularis*, though overlapped in distributions.

Most notably, the ratio of eye width to head width was very distinct in this group, with relatively “very small eyes”. This should be considered a diagnostic feature in these frogs, with ratios of ~0.35 for *H*. *ukaguruensis* sp. nov. compared to ~0.55–0.60 for all other species.

In coloration, *H*. *ukaguruensis* sp. nov. has similarities in dorsal patterning to other members of the complex in that individuals have a greenish-brown uniform coloration with either mottled white patterns on the dorsum or distinct white dorsolateral lines extending from a canthal triangle on the snout reaching nearly the groin. Dorsolateral lines contain small black speckles. *Hyperolius ukaguruensis* sp. nov. has distinct dorsal coloration of this uniform color, however, from other spiny-throated reed frogs. *Hyperolius ukaguruensis* sp. nov. has golden-green dorsal coloration with flashes of orange on the thighs and feet. *Hyperolius spinigularis*, *H*. *burgessi*, *H*. *davenporti*, and *H*. *tanneri* have silvery-green tones in their dorsal coloration. *Hyperolius ruvuensis* also has a brown dorsal color, and has a cream ventral surface and orange flashes on the thighs. *Hyperolius ukwiva* is described as brown in dorsal coloration, though lack of photos and live specimens makes it difficult to quantify coloration variation. *Hyperolius minutissimus* individuals also have distinct coloration from other species in this clade, with mottled yellows and green similar to *H*. *pictus* coloration, but which do not resemble *H*. *ukaguruensis* sp. nov.

Ventral coloration is also distinct in *H*. *ukaguruensis* sp. nov., which is bright yellow on many individuals in life. Only *H*. *ukwiva* also has a yellow ventral surface.

Based on molecular comparisons, *H*. *ukaguruensis* sp. nov. is also genetically distinct from close relatives, and is minimally 2.9% pairwise divergent from its closest relative (*H*. *ukwiva*), based on mtDNA (Table 2; see Fig 2) with unique nuclear haplotypes. *Hyperolius ukaguruensis* sp. nov. also has an allopatric distribution with respect to all other species in the complex (Fig 1).

*Description of holotype*.—Small sized hyperoliid. Horizontal pupil. The snout is blunt and slightly rounded. See Greenwood et al. 2020, Fig 2 for visualization of measurements. Canthus rostralis is angular, being slightly convex on the horizontal plane and slightly concave on the vertical plane. Snout-urostyle length is 21.5 mm and head width is 7.1 mm (33% of SUL). Head length (diagonal to the corner of the mouth) is 6.9 mm, and diagonal to the jawbone is 7.0 mm. Distance between the eyes is 3.0 mm and the inter orbital distance is 4.3 mm. The inter-narial distance is 2.3 mm and the narial distance to the eye is 2.0 mm. The nostril to snout is 1.7 mm. Gular disc width is 6.8 mm and the height is 5.4 mm. Tibio-tarsal articulation of the adpressed hind limb reaching past the eye. Crus length (Tibiofibula bones) (10.8 mm) is greater than thigh length (8.8 mm). The tarsus length (Tibiale Fibulare bones) is 5.8 mm. Foot length is 9.7 mm. The toes have expanded fleshy discs. Webbing on the toes is moderate, reaching the base of the fleshy discs on all toes apart from the first toe where it only reaches the first tubercle. The forelimb length is 5.2 mm, and the hand length is 6.6 mm. The hands have expanded, rounded fleshy discs. The Webbing just reaches the distal subarticular tubercle of the outer finger, and reaches the distal subarticular tubercle of the 4th finger on both sides. Dorsal skin surface is granular. Ventral skin surface is granular with asperities, with sparse asperities present on mid-ventral region near the midline and on the groin and thighs. Asperities on the gular flap are concentrated medially and anteriorly, with additional spines along the bottom of the lower jaw (Fig 5). Dorsal coloration is green/gold/tan with white/cream mottling on the dorsal surface extending from a triangle on the snout along the dorsum to near the urostyle. Toes are golden yellow with red thighs, and the ventrum is translucent with hints of yellow patches.

*Paratypes*.—Head and body proportions are in close agreement with those of the holotype (see Fig 4, Table 3, S3 File). The distribution of the asperities of other males are medially and anteriorly concentrated on the gular flap with sparse spines along the mid-ventral region, groin and thighs. The number of spines varies. The proportions of the gular flap, diagnostic for *H*. *ukaguruensis* sp. nov., is greater in width than height, and rounded with a slight bilobed shape (Figs 4 & 5). Webbing of all the material conforms to that of the holotype. Females have no ventral spines and have more granular ventra. Some of the paratypes did not share the mottled dorsal patterns of the holotype, instead showing a broad dorsolateral stripe running from the tip of the snout, over the eyelids to the inguinal region. The stripe is white or cream in color, mottled with darker spots or blodges. Both phenotypes were seen in males and females. Ventrum color is also variable with some individuals showing completely yellow coloration, though the degree of yellow clearly varies.

Paratypes BMNH 2022.7683, and field numbers SL_4057 and SL_4058 showed distended abdomens and translucent parts of the skin showed white ova with a pigmented pole, suggesting females breeding during this time of the year.

**Fig 7:**
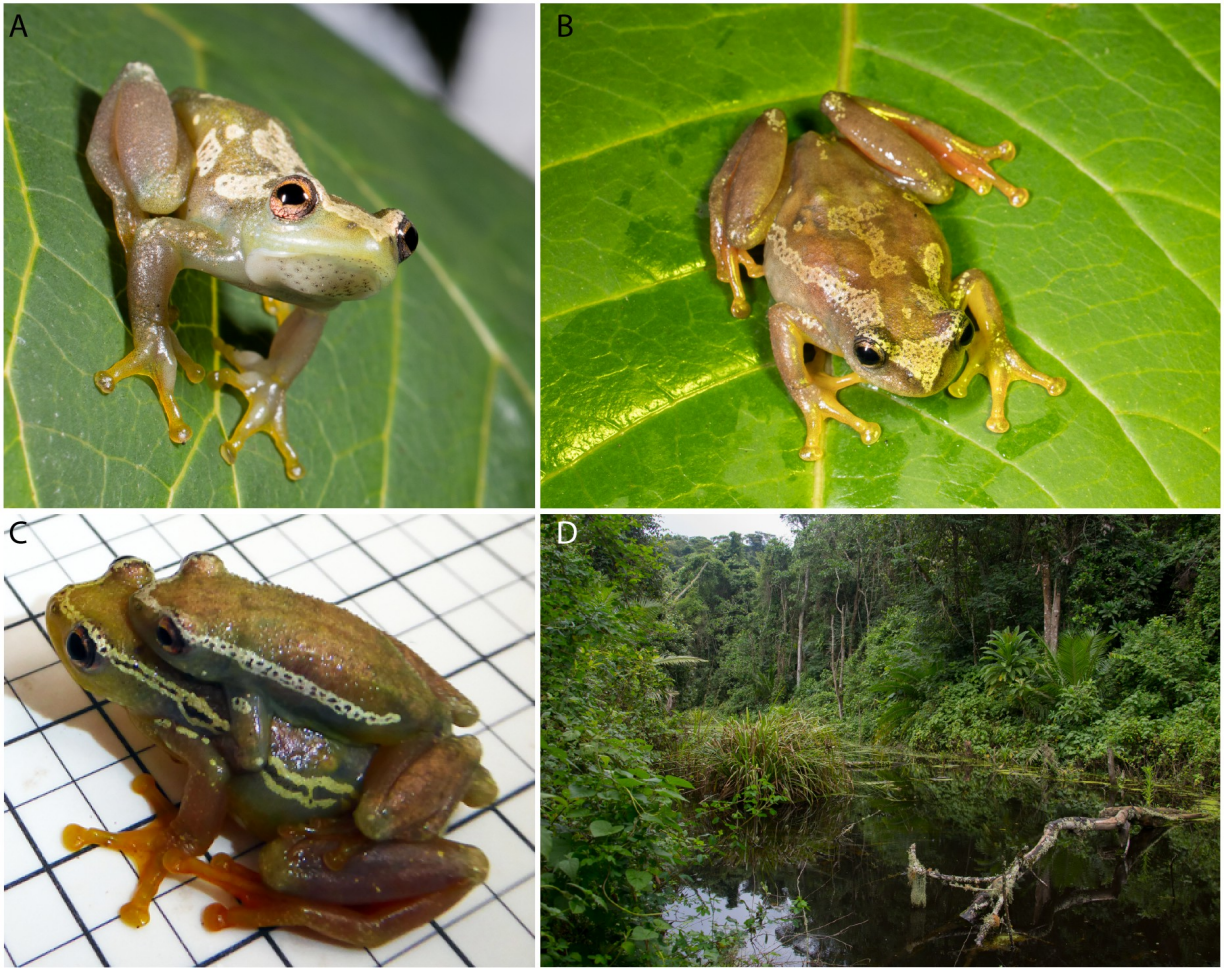
Photos in life of *H*. *ukaguruensis* sp. nov. A,B) Male (BMNH 2022.7682; holotype) and female (BMNH 2022.7683; paratype) *in vivo*, C) *Hyperolius ukaguruensis* sp. nov. male and female in axillary amplexus, and D) Type locality habitat. Photos by C. Liedtke.

*Color patterning of adults in life*.—See Figs 6 and 7 for photo in life. Generally, the females and males resemble the holotype in coloration. The dorsum is described in field notes as being “brown with two light cream-brown stripes from nose to the hindlegs” for most specimens with “silvery-gold mottling” on other specimens (including the holotype). Overall, the hue is much more “golden” in *H*. *ukaguruensis* sp. nov. than most spiny-throated reed frogs which tend to have cooler greens, with silver and white markings for their coloration. The variation between some individuals with dorsolateral stripes and some individuals with silvery mottling is also common within the spiny-throated reed frogs (see photos at amphibiaweb.org and in [3]). The ventrum is sunshine yellow, which is distinctly different from other frogs in this clade except the closely related *H*. *ukwiva*, which tend to have white or cool green ventral coloration. The legs and arms are similarly colored to each other, both dorsally and ventrally, with bright red thighs and underarms.

*Sexual dimorphism*.—Females attain a much larger size than the males (Figs 3 & 6). Asperities of the dorsum are weaker in females and absent from the ventral side in females. Males are easily distinguished from the females during the breeding season by their characteristic rounded and wide gular sac (Fig 6).

*Advertisement Call*.—No calls were detected or recorded during collection of the type series.

*Etymology*.*—Hyperolius ukaguruensis* sp. nov. is named after the forested mountain block (Ukaguru Mountains) where the type series was collected. The species name is a masculine Latin singular adjective in the nominative case.

*Distribution*, *Ecology and Conservation*.*—Hyperolius ukaguruensis* sp. nov. is only known from one locality in Mamiwa Kisara North Forest Reserve in the Ukaguru Mountains. Specimens were collected in and around the edge of montane forest in a swamp. Collecting across the Ukaguru Mountains has been patchy [9], so it is not clear if *H*. *ukaguruensis* sp. nov. has a localized distribution. Unfortunately, Mamiwa Kisara North Forest Reserve is impacted by anthropogenic impacts–including deforestation. Forest loss has recently been exacerbated by fire which has impacted large patches of forest. Given its small distribution and habitat loss, *Hyperolius ukaguruensis* sp. nov. is likely to be of high conservation concern [9].

## Discussion

The spiny-throated reed frogs show substantial population structure between geographically restricted areas in Tanzania, Malawi, and Mozambique. These species are narrowly distributed endemics (Fig 1), mostly restricted to either single mountain blocks (e.g., West Usambara Mountains) or a few nearby mountainous areas (e.g., East Usambara Mountains, Nguru/Nguu Mountains, and Uluguru Mountains). The only exception to the montane distribution is the recently described species from a small patch of forest in lowland coastal areas (*Hyperolius ruvuensis*, Ruvu South Forest) [1, 2]. Each species has significant genetic divergence from all other lineages, distinct morphology, and different patterns and extent of the gular asperites that typify this group.

Other co-distributed *Hyperolius* species do not appear to show as much single-mountain block endemism within the Eastern Arc Mountains and nearby mountain blocks [7, 38–40]. Two characteristics of this clade may play a role in the increased tendency towards allopatric speciation in this clade compared to co-distributed congenerics. One explanation is that this clade is considered voiceless (or “nearly voiceless”) with no known calling behavior and only small raspy sound emitted within a plastic bag for *H*. *minutissimus* [8]. This may limit their ability to expand their ranges and find new mates in new environments if they cannot transmit their presence over large areas as other *Hyperolius* males can when they call at night. Second, these species appear to be habitat specialists, found in dense to semi-dense concentrations in some areas with in-tact habitat. This includes Amani Nature Reserve in the East Usambara Mountains of Tanzania with closed canopy primary forest cover and deep and stable wetlands and not marginal habitats or more seasonal water bodies as seen in other co-distributed species *H*. *substriatus*, *H*. *mitchelli*, and *H*. *rubrovermiculatus* [11, 38, 41]. If each species is a habitat specialist adapted to their specific localities, gene flow between regions will be severely limited even during shifting historical climate cycles thought to link populations of other *Hyperolius* species [11].

The only exception we found to this pattern of highly divergent lineages on different mountain blocks are the sister species *H*. *burgessi* and *H*. *davenporti* found in Northern and Southern Tanzania (Fig 1). These two lineages are morphologically distinguishable (Figs 4 & 5), yet the genetic distances from the mitochondrial and nuclear loci are small. Evidence supports *H*. *davenporti* as being distinct given its distinct morphological characteristics, molecular differences and geographical isolation, but it might be a “newly evolved species” as outlined in other recent examples in the region [42].

*Hyperolius ukaguruensis* sp. nov. is noticeably distinct in morphology, coloration, genetics, and distribution from all other known species in this group. Combining interpretations from our genetic and genomic analyses, we anticipate that *H*. *ukwiva* would be the sister species to *H*. *ukaguruensis* sp. nov. (Fig 2). This is not unexpected, as the Rubeho Mountains are ~80 km from the Ukaguru Mountains. These lineages are distinct, however, in mitochondrial divergence (2.9%), unique nuclear haplotypes in line with other lineages (POMC and RAG1), allopatric distribution, and morphology (female eye width/head width ratio as the best indicator). Further surveys of *H*. *ukwiva* are needed to better compare similarities in males and gain genomic data. Both of these species have only been found in one single waterway, despite surveys throughout these mountain blocks. Further surveys throughout this region will also help clarify if additional populations exist or if they are truly restricted to extremely small areas. Comparison of occurrence of endemic and regional endemic Eastern Arc Mountains forest vertebrate species demonstrated close affiliation of Ukaguru and Rubeho mountains [43] and our study shows supporting phylogenetic evidence to this finding.

A second new lineage was revealed in these analyses, within *H*. *minutissimus* populations. Differences between populations of *H*. *minutissimus* in the Udzungwa Mountain block were previously suspected based on morphology (personal observations, LPL, SPL, M. Menegon, D. Moyer) and the fact that Schiøtz proposed a lineage of *H*. *spinigularis* and a lineage of *H*. *minutissimus* within the block [8], which we propose are actually two lineages of *H*. *minutissimus*. Samples from Sao Hill (newly included in this paper) and the Uzungwa Scarp population (previously sampled in [1]) are genetically divergent (3.3% divergence based on 16S data). This large divergence is greater than differences between other known described species in the spiny-throated reed frog complex (see [1], Table 2, and Figs 2 & 3). The type locality for *H*. *minutissimus*, in Njombe Tanzania, is ~80 km from the Sao Hill locality and ~150 km from the locality from the Uzungwa scarp. Specimens from Idete (~215 km from the type locality of *H*. *minutissimus*) and Massisiwe village and nearby Uzungwa Scarp were classified as *H*. *spinigularis* in [8], whereas Sao Hill collections were classified as *H*. *minutissimus*. These descriptions, in reference to the specimens from the type locality suggest that the Sao Hill collection may reflect true *H*. *minutissimus* and the specimens from the Uzungwa scarp represent a new lineage yet to be named. Detailed genetic and morphological evaluations of these lineages are required.

Lawson *et al*.’s [2] “genetic” analyses were able to resolve the “tips” of the tree to establish sister relationships, however deeper nodes were too poorly resolved to understand speciation within this lineage. In this study, genomic analyses based on a highly filtered SNP dataset revealed clear phylogenetic relationships mirroring the topology in [19] where *H*. *tanneri* was basal to *H*. *spinigularis* and *H*. *minutissimus*. Portik *et al*.’s [19] analysis only included four species and not the known diversity outlined here, but our study now clarifies the relationships within this radiation. The STRUCTURE analyses showed *Hyperolius tanneri* as containing loci from both the “spinigularis” and “minutissimus” clades (under K = 3), which might explain why resolving the relationships between the groups has been historically challenging. As we do not expect gene flow between these distinct lineages, this may reflect the short branching times when all three major lineages split. Further clusters (K = 4 and 5) did not show this result, though the implication of a nearly simultaneous split of the three main lineages may mean that incomplete lineage sorting is still in effect despite significant time passing.

Confirming the placement of *H*. *tanneri* as potentially basal or near basal to the rest of the clade might help clarify the role that the gular spines and lack of “voice” play in evolution within this clade. These are both highly unusual traits for *Hyperolius* frogs (or any frogs). If *H*. *tanneri* is ultimately basal with *H*. *ukaguruensis* sp. nov. and *H*. *ruvuensis* as the next lineage in the phylogenetic tree, then the spine-free throat of *H*. *tanneri* matched with the voiceless nature of those frogs, would imply that loss of calling occurred first, followed by the subsequent synapomorphy of spines on the throat (and in some cases, chest and groin) of other species. Of note, *H*. *minutissimus* has a very quiet and high pitched call, which was able to be recorded in a tent [4], but is unlike essentially all other *Hyperolius* species which have an easily audible call. The cause of a “non-calling” or “quiet” trait in this group of frogs may not be known, or even fully characterized (*H*. *spinigularis*/*H*. *davenporti*/*H*. *tanneri* have been heard making small sounds in a bag, though these do not seem to be mating calls [4, 7]), but the secondary acquisition of spines may play a role in species recognition to fit a clade of silent and secretive frogs through enhanced pheromone delivery [44–46].

The spiny-throated reed frogs appear especially vulnerable to extinction threat, even for amphibians which are generally declining, due to the combination of being habitat specialists, single locality endemics, and possessing small population sizes [47]. For instance, *H*. *ruvuensis*, was recently described from a single coastal forest in Tanzania yet has not been encountered since 2001 [1]. This new lineage, *H*. *ukaguruensis*, also appears to be unique, a habitat specialist, and to have a very narrow range. In naming this *H*. *ukaguruensis* sp. nov., we hope to bring awareness to the value of additional biodiversity surveys and careful taxonomic consideration in East Africa to identify the full extent of lineages found there. This is especially critical during the current amphibian extinction crisis of combined threats of habitat destruction, climate change, and chytrid fungus infections [48].

## Supporting information

S1 File Summary information of specimens included for molecular analyses, localities, and NCBI GenBank accession numbers for Sanger sequenced loci and ddRAD submissions. (XLSX)

S2 File Phylip file of 1909 ddRAD SNPs for genomic dataset. (TXT)

S3 File Measurements, notes, location, and museum IDs for all samples included in morphological analyses. All measurements are recorded in cm.
(XLSX)

S4 File PCAs loadings of the male morphological datasets showing loadings for each Principle Component. (XLSX)
